# Role of frailty on cardiac rehabilitation in hospitalized older patients

**DOI:** 10.1007/s40520-022-02220-x

**Published:** 2022-09-05

**Authors:** Leonardo Bencivenga, Grazia Daniela Femminella, Pasquale Ambrosino, Quirino Bosco, Claudio De Lucia, Giovanni Perrotta, Roberto Formisano, Klara Komici, Dino Franco Vitale, Nicola Ferrara, Mauro Maniscalco, Francesco Cacciatore, Antimo Papa, Giuseppe Rengo

**Affiliations:** 1 Department of Advanced Biomedical Sciences, University of Naples “Federico II”, Naples, Italy; 2 Gérontopôle de Toulouse, Institut du Vieillissement, CHU de Toulouse, Toulouse, France; 3 Department of Translational Medical Sciences, University of Naples “Federico II”, Naples, Italy; 4 Cardiac Rehabilitation Unit, Istituti Clinici Scientifici Maugeri IRCCS, Scientific Institute of Telese Terme, Bagni Vecchi, 1, 82037 Telese Terme, Italy; 5 Department of Medicine and Health Sciences, University of Molise, 86100 Campobasso, Italy; 6 Clinica San Michele, Via Appia 187, 81024 Maddaloni, CE Italy

**Keywords:** Frailty Index, Cardiac rehabilitation, Comprehensive geriatric assessment, 6-min walk test

## Abstract

**Background:**

Cardiovascular diseases are the leading cause of mortality, morbidity, and disability in the world, especially in the older adults. A relevant proportion of patients admitted to Cardiac Rehabilitation (CR) may suffer from frailty, a complex geriatric syndrome with multifactorial aetiology.

**Aims:**

The hypothesis underlying the study is that frailty complicates the management of older patients undergoing CR. The main objective is, therefore, to determine the relationship between frailty and CR outcomes in hospitalized older adults.

**Methods:**

The participants have been recruited among patients aged ≥ 65 years admitted at the hospital for CR. A Comprehensive Geriatric Assessment (CGA)-based Frailty Index (FI) was created following a standard procedure. The outcome was measured as the ratio between 6-min walk test (6MWT) distance at the end of CR and normal predicted values for a healthy adult of same age and gender, according to reference equations.

**Results:**

The study population consisted of 559 elderly patients, 387 males (69.2%), with age of 72 (69–76) years. The most frequent diagnosis at admission was ischaemic heart disease (231, 41.5%) and overall 6MWT ratio was 0.62 ± 0.21. At the multivariable regression analysis, gender, diagnosis and FI were significantly and independently associated with 6MWT ratio (*p* ≤ 0.0001, *p* ≤ 0.001 and *p* ≤ 0.0001, respectively), while no significant association emerged for age.

**Conclusion:**

FI resulted independently correlated to 6MWT ratio in a population of older patients undergoing in-hospital CR programs. Frailty is a multifactorial geriatric syndrome whose assessment is essential for prognostic evaluation of older patients, also in CR clinical setting.

**Supplementary Information:**

The online version contains supplementary material available at 10.1007/s40520-022-02220-x.

## Introduction

At the beginning of 2019, there were 90.5 million people aged 65 years or more living in the European Union, and the number is expected to peak 129.8 million people in 2050, when the older adults will constitute approximately 30% of all European citizens [1]. The progressive aging of the population is a worldwide phenomenon, and it is accompanied by significant social, economic and health issues. Indeed, with increasing age, the risks of developing chronic disease and care dependency increase [2].

One of the most problematic expressions of population aging is frailty, a multifactorial condition of increased vulnerability and poor resolution after a stressor event, due to reduced homeostasis. It is associated with an increased risk of adverse outcomes, such as disability, falls, long-term care and death [3]. Approximately 25–30% of people older than 85 are reported to be frail, therefore the assessment of frailty should be an essential part of the clinical work-up of the older patient. Two main models of frailty have been proposed: the frailty phenotype by Fried and collaborators, which asserts that this condition can be described through the measurement of five physical features (unintentional weight loss, weakness, exhaustion, slow walking speed and low physical activity) [4], and the cumulative deficit model, advanced by Rockwood et colleagues, which views frailty as the proportion of potential deficits which are present in an individual [5]. Although both models show good overlap at the identification of frailty, the continuous variable frailty index (FI) deriving from accumulation of deficit model, shows better discrimination for people with moderate and severe frailty compared with the categorical phenotype model [6]. Furthermore, several evidence underlined that the different discriminatory power of the various employed instruments can determine discrepancies in the identification of frailty subtypes, with implications on prevention and treatment strategies [7].

According to recent data, cardiovascular diseases (CVD) represent the leading cause of morbidity, disability, loss of physical functioning and mortality in people aged over 50 years worldwide [8]. In the United States, the overall prevalence of CVD, comprising ischemic heart disease (IHD), heart failure (HF), stroke, and hypertension, is 48% in adults  ≥ 20 years old and increases with age in both males and females. CVD currently claim more lives each year than cancer and chronic lung disease combined [9]. Cardiac rehabilitation (CR) is a comprehensive, multidisciplinary intervention, tailored on each single patient with CVD, which includes exercise training programs, lifestyle modification, and psychological support. It has been shown to improve exercise tolerance, patients’ well-being and quality of life, and to reduce the risk of new cardiac events [10]. Interestingly, patients older than 75 years represent about one-third of those referred to CR [10], therefore, frailty might be present in a large proportion of patients undergoing CR, but this aspect has not been thoroughly tested so far.

In this research, we aimed to investigate whether multidimensional frailty might complicate the management of older patients undergoing CR. The main objective of the study is, therefore, to determine the relationship between frailty and CR outcomes in hospitalized older adults.

﻿Method﻿s

### Study population

The participants have been recruited among patients referring to Istituti Clinici Scientifici Maugeri IRCCS, Telese Terme Institute, Italy for CR after HF exacerbation, IHD, valvular heart diseases (VHD), cardio-aortic surgery, and other cardiovascular conditions. Patients were excluded if they refused to sign informed consent or if they had any condition associated with poor compliance with the study protocol.

At admission, all patients underwent medical history collection, clinical examination and evaluation of the main demographic/clinical factors. The research protocol was reviewed and approved by the Local Ethics Committee (reference number ICSM/17/1).

### Frailty assessment

At the time of enrollment, all stable patients underwent Comprehensive Geriatric Assessment (CGA), with the evaluation of the domains of health and functional status, psycho-cognition, socio-environmental condition.

A CGA-based FI was adapted from the standard procedure proposed by Rockwood’s research group, taking into account a total of 40 multidimensional health deficits including comorbidities, laboratory and diagnostic data, symptoms and sign of diseases (Supplemental Tables 1 and 2) [11]. Each deficit was awarded 1 point if present, or 0 in its absence. FI for single participant resulted by the ratio between her/his cumulative points and the total number of evaluated items, thus this ranged between 0 and 1. A cut-off of 0.25 was applied to define participants as frail (FI ≥ 0.25) and non-frail (FI < 0.25).

**Table 1:** Characteristics of the overall population and of subgroup according to frailty status

Characteristics	All patients (*n* = 559)	Non Frail (*n* = 266)	Frail (*n* = 293)	sig *p* value
Age (years)	72 (69–76)	71 (68–75)	73 (69–77)	< 0.001
Gender (male)	387 (69.2)	181 (68)	206 (70.3)	0.563
BMI (Kg/m^2^)	28 (25–30.9)	27.05 (24.8–29.8)	28.8 (25.6–32)	< 0.001
MMSE	24 (20–28)	25 (21–29)	23 (18–27)	< 0.001
GDS	2 (1–4)	1 (1–3)	3 (1–5)	< 0.001
BADL	6 (6–6)	6 (6–6)	6 (6–6)	< 0.001
IADL	5 (4–7)	6 (4–7)	4 (3–5)	< 0.001
MNA	25 (23.5–26)	25.5 (24.5–26)	25 (23–26)	< 0.001
CIRS-C	5 (4–7)	5 (3–7)	6 (5–7)	< 0.001
CIRS-S	2.2 (1.9–2.5)	2 (1.8–2.3)	2.2 (2–2.5)	< 0.001
Number of drugs	9 (8–11)	8 (7–10)	10 (8–12)	< 0.001
PASE	81 (41–121)	95 (70–126)	61 (20–106)	< 0.001
Social support score	6 (4–7)	5 (4–7)	6 (5–8)	< 0.001
Exton-smith	18 (17–19)	18 (18–19)	18 (16–19)	< 0.001
SPPB	11 (8–12)	11 (9–12)	10 (7–12)	< 0.001
POMA	28 (25–28)	28 (26–28)	28 (24–28)	0.003
Frailty Index	0.25 (0.19–0.3)	0.19 (0.16–0.22)	0.3 (0.26–0.33)	< 0.001
Diagnosis at admission				
Heart failure	138 (24.7)	46 (33.3)	92 (66.7)	< 0.001
Ischaemic heart disease	232 (41.5)	105 (45.3)	127 (54.7)	
Valvular heart disease	166 (29.7)	97 (58.4)	69 (41.6)	
Other CV conditions	23 (4.1)	18 (78.2)	5 (21.8)	
6MWT (m)	275 (203–348)	300 (236–360)	260 (180–330)	< 0.001
Predicted 6MWT (m)	440.54 ± 57.42	451.5 ± 54.05	430.59 ± 58.65	< 0.001
6MWT ratio	0.62 ± 0.21	0.66 ± 0.21	0.58 ± 0.21	< 0.001

The *p* value corresponds to Student’s *t* test or Mann–Whitney *U* test for continues variables, and chi square test for categorical data

6MWT, 6-min walking test; *GDS* Geriatric depression scale, *BADL* Basic activity of daily living; *BMI* Body mass index, *CIRS* Cumulative illness rating scale, *CV* cardiovascular, *IADL* Instrumental activity of daily living, *MMSE* Mini mental state examination *MNA* Mini Nutritional assessment; *PASE* Physical activity scale for the elderly, *POMA* Tinetti’s Performance oriented mobility assessment, *SD* Standard deviation, *SPPB* Short performance physical battery

**Table 2:** Regression analysis for 6MWT ratio

6MWT ratio R^2a^: 0.226			
Variables	B	SE	Sig
Age	− 0.024	0.001	0.118
Gender (male)	0.166	0.017	< 0.0001
Diagnosis	− 0.036	0.01	< 0.001
Frailty Index	− 0.666	0.099	< 0.0001

### Six-minute walking test

At the end of CR, all patients underwent the 6-min walking test (6MWT) following indications provided by American Thoracic Society guidelines [12]. It was performed by trained physicians who monitored the procedure and invited subjects to walk at their own maximal pace on a 20-m long hospital corridor, after 30 min of absolute rest from physical activity.

Predicted 6MWT was determined for each participant through reference equations for both genders based on patients’ age, height and weight, while the 6MWT ratio was measured as the ratio between 6MWT distance and normal predicted values, as previously described [13–16].

### Statistical analysis

Continuous variables were expressed as mean ± Standard Deviation (SD) or median and interquartile range (IQR) and compared using Student's *t* test or Mann–Whitney *U* test. Normal distribution was assessed using the Shapiro–Wilk test. The categorical variables were expressed as absolute observation and percentage and compared using the *χ*^2^ test. Multivariable regression analysis was used to identify factors associated with 6MWT ratio. Parsimonious selection criteria were used to avoid overfitting bias, also taking into account the multidimensional nature of CGA-based frailty assessment: the analysis considered age, sex, FI and diagnosis at admission as independent variables.

All analyzes were performed using a *p* < 0.05 as a statistical significance threshold, through the Stata17 software (StataCorp, Lakeway Drive, Texas 77,845 USA).

## Results

**Fig. 1:**
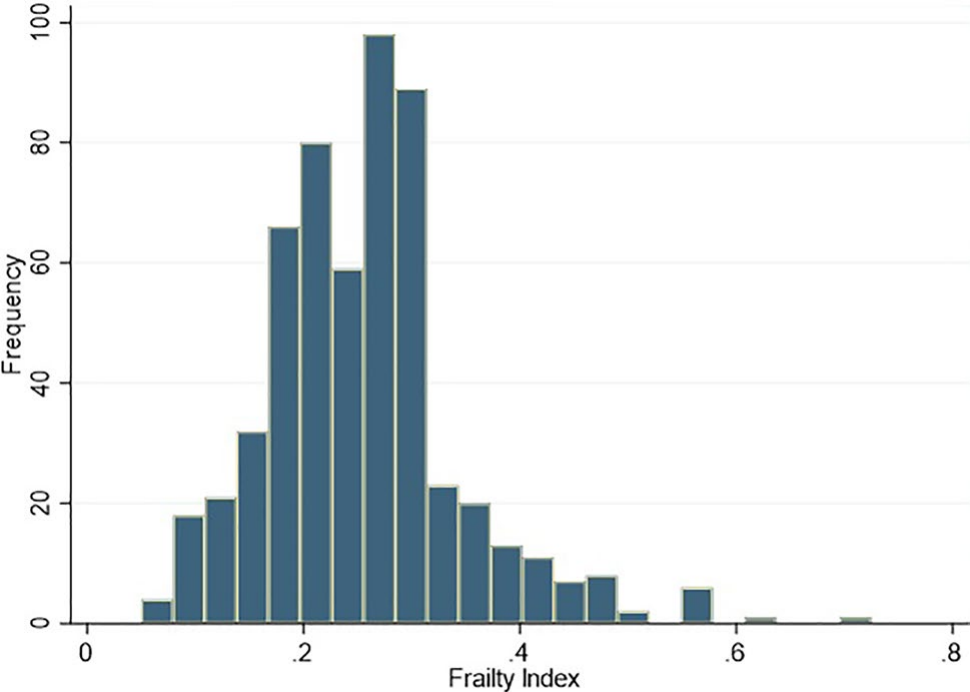
Frailty Index distribution in the study population

**Fig. 2:**
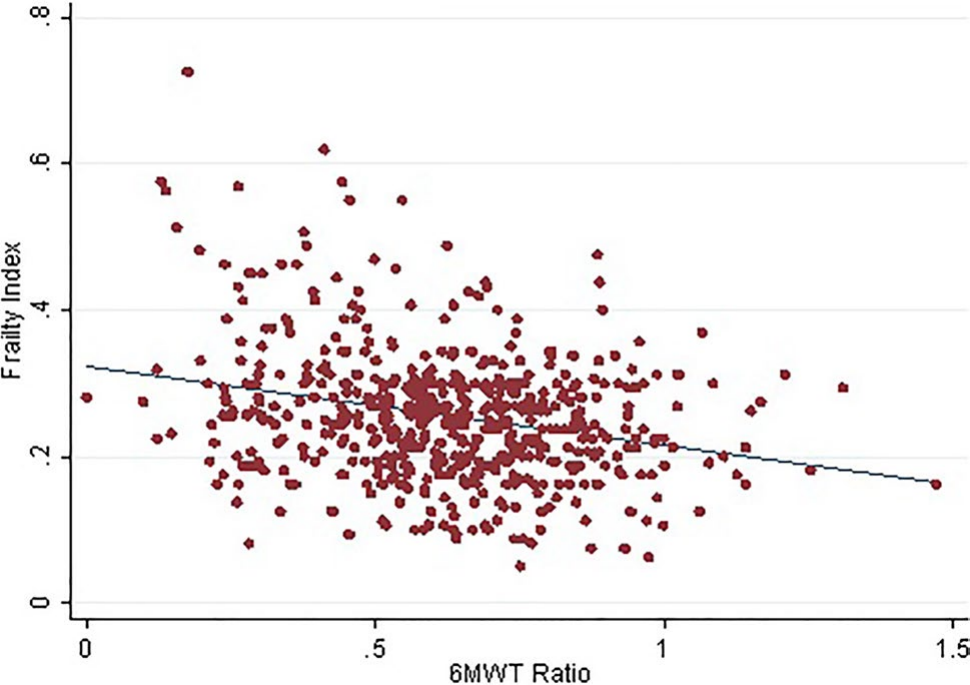
Scatter plot for 6MWT ratio and Frailty Index in the study population

The study population consisted of 559 older adults, mostly males (387, 69.2%), with median age of 72 (69–76) years and Body Mass Index (BMI) of 28 (25–30.9) Kg/m^2^. The patients mainly accessed the CR programs offered by the clinic for recovery from IHD (231, 41.5%), HF exacerbation (138, 24.7%), and correction of VHD (166, 29.7%). A minority of patients (23, 4.1%) were hospitalized for other cardiovascular conditions, such as cardio-thoracic surgery and myxoma. Data on the global sample and on groups stratified according to frailty status are reported in Table 1. At enrollment, participants presented a FI of 0.25 (0.19–0.3) (Fig. 1), whereas the predicted 6MWT and the 6MWT distance performed at the end of CR were 440.54 ± 57.42 and 275 (203–348) m, respectively, accounting for an overall 6MWT ratio of 0.62 ± 0.21. The relationship between FI and 6MWT ratio is shown in Fig. 2.

Frail patients were slightly more numerous than non-frail ones (293, 52.4% vs. 266, 47.6%) and significantly older; at univariate analyses, they also presented higher BMI, while no differences emerged in terms of gender. The frailer group presented worse score in all the CGA domains, as demonstrated by results in tests and scales exploring dependency [Activity of Daily Living (ADL), Instrumental Activity of Daily Living (IADL)], phyco-cognition [Mini Mental State Examination (MMSE), Geriatric Depression Scale (GDS)], comorbidities [Cumulative Illness Rating Scale (CIRS)], nutrition [Mini Nutritional Assessment (MNA)], polypharmacotherapy, global status (Exton-Smith, Social Support Scale), and physical performance [Physical Activity Scale for the Elderly (PASE), Short Performance Physical Battery (SPPB), Tinetti’s Performance Oriented Mobility Assessment (POMA)].

At multivariable regression analysis for 6MWT ratio, the model included as independent variables age, gender, FI and diagnosis at admission and explained 23% of dependent variable variance in the study population (Table 2). The analysis revealed the 6MWT ratio to be significantly and independently associated with male gender, diagnosis and FI (*p* < 0.0001, *p* < 0.001 and *p* < 0.0001, respectively), while no relevant association emerged with chronological age.

## Discussion

The present manuscript addresses the relevant clinical issue of the role exerted by frailty on CR programmes in hospitalized older adults. The main result is that general frailty, intended as multidisciplinary cumulative deficit assessed through CGA-based FI, an accurate estimate of vulnerability, independently impacts the 6MWT ratio, a CR outcome very indicative of the potential of each individual participant. Although many pieces of literature have previously analyzed the relationship between frailty models and CR outcomes, to the best of our knowledge, this is the first study exploring the impact of multidimensional cumulative decline frailty at admission on CR intervention in older CVD patients.

Within the great progresses achieved in CVD management over the last 20 years, CR has relevantly concurred to ameliorate prognosis, prevent disability and positively influence the quality of life of afflicted patients and their caregivers [10, 17]. Nevertheless, the potential for significant health benefits in older people remains indecipherable, despite recommendations of guidelines for CR programmes participation [18]. Indeed, even if multimorbid older adults represent a considerable proportion of patients involved in CR in the last decades [19], the conceptualization of frailty is comparatively recent, therefore, large CR trials leading to current evidence were not intended to address this complex geriatric syndrome [20]. Furthermore, multiple concomitant conditions often represented exclusion criteria in CR trials, thus determining the absence of older patients [21]. Notably, the paradoxical consequent effect is that specific evidence on CR efficacy is limited for frail older adults, although they represent the target population that would potentially benefit most from this intervention [22]. Accordingly, it has been advised to include frailty assessment as a key component in the clinical work-up of these patients [23], with the paradigmatic example constituted by the Heart Team multidisciplinary approach to the complex patient with VHD [24]. Similarly, in older patients with acute coronary syndrome, frailty has been independently associated with increased comorbidity and longer hospital stay, with the highest frailty category representing a negative prognostic factor [25]. Moreover, in older patients with non-ST-segment elevation myocardial infarction (NSTEMI), frailty has been found to independently relate with 1-year mortality, after adjusting for cardiovascular risk and comorbid conditions [26] and has demonstrated added predictive value on top of standard vascular risk scores in these patients [27]. Frailty status showed independent prognostic value also in the clinical setting of older adults with HF [28, 29], thus confirming the usefulness of incorporating its assessment into the clinical management of these patients.

In recent years, in the context of CR, several evidence have explored the association between rehabilitative programs and frailty indicators, but the topic is still burdened by an excessive heterogeneity of outcome measures and methods for assessing the global status of enrolled patients. The multicentre, randomized, controlled REHAB-HF trial compared multiple physical-function domains intervention versus usual care in 349 older patients hospitalized for acute HF. After 3 months, the intervention group showed great improvement in physical frailty, assessed through the primary outcome (SPPB); a modified frailty phenotype was also employed as secondary outcome to test for improvement of global status, revealing the frail subgroup to benefit more from the CR program than the non-frail or prefrail ones [30]. A retrospective analysis on 51 Brazilian patients aged ≥ 65 years undergoing CR program revealed a significant improvement in the frailty status of participants, assessed through the Edmonton Frail Scale (EFS) at the beginning and 3 months after intervention, with the frailer ones experiencing the best improvement of global status [31]. Kehler and collaborators performed a secondary analysis on 4004 CVD adults referred to CR clinic for 12-week program, measuring frailty at baseline and at the end of intervention through a 25-item FI; the authors reported a significant reduction in frailty status at the end of the rehabilitation, especially in patients with severe frailty. Very interestingly, they also highlighted how higher FI was associated with CR drop-out [32], thus stimulating the debate on the role of frailty as a modulator of intervention programs.

As matter of the fact, conventional CR generally comprises aerobic and resistance training, which can be excessively challenging for frail patients, whose clinical complexity is determined by multiple concomitant factors that go beyond the more intuitive possible impairment in musculoskeletal and physical functions. Indeed, several pieces of literature have investigated the role of other phenotypic domains of aging on predicting successful CR: total energy intake and nutritional status assessed through MNA-Short Form score at the beginning of in-hospital rehabilitative intervention predicted final functional dependence in 145 older HF patients [33]. Moreover, Rao and colleagues demonstrated the relevance of depression and anxiety in a retrospective cohort of nearly 6000 patients entering CR, with those suffering from these conditions being less likely to adhere the planned training programs [34]. A comparable negative modulating effect on the effectiveness of CR programs is exerted by another tremendous burden of aged patients, represented by psychosocial aspects, as social isolation and low socio-economic status [35]. The Cardiac Rehabilitation Section of the European Association of Cardiovascular Prevention and Rehabilitation of the European Society of Cardiology recently published a position paper to encourage the inclusion of a multimodal behavioral intervention within the CR, with the aim of assessing and addressing the socio-economic domain in afferent patients [36].

The present manuscript is inserted in the wake of this evidence, with the objective of evaluating the impact of multidimensional frailty, including the CGA-based assessment of all the main phenotypic dimensions that characterize the aging process in addition to physical impairment, on the rehabilitation outcome of older patients suffering from CVD. It is worth mentioning that the discordance among different models and classifications of frailty pave the stimulating discussion about the real meaning of this complex condition, from syndromic manifestations of vulnerability to the broad concept of cumulative multidomain deficits, including impairment, disability, and comorbidity [7]. Accordingly, taking into account that the definition of frailty employed in the present research incorporates disability as an item to calculate the score rather than as an outcome, our results are particularly interesting, because they show how the robust FI, obtained through 40 multidimensional age-related items, is significantly correlated to the outcome of CR in a population of older adults with CVD. Notably, it is important to underline that age, although significantly higher in the frail group than in non-frail patients, when included in the final model together with frailty, does not significantly impact the 6MWT ratio. This indicates that the cumulative multidimensional decline, measured by the FI, is better at predicting post-rehabilitation physical performance, even compared to chronological age, which constitutes an intrinsic characteristic of aging.

If confirmed in other studies, the CGA-based FI should be assessed in older patients at the admission in CR to carry out a holistic assessment and to let physicians detect and address impairments in geriatric multidimensional domains before beginning exercise programs. This procedure would allow to facilitate and ameliorate participation of frail people to CR program, to improve personalized interventions and to optimize healthcare resource allocation.

### Strengths and limitations

In addition to the robust model used to assess frailty and the evidence of the superiority of the “biological” age over the “chronological” one in predicting the outcome, two other points of strength of the present work are the high sample size, since it is a population of almost 600 older adults referred to a rehabilitation clinic for CVD, and the rehabilitation outcome employed itself. Indeed, the 6MWT ratio is adjusted for the theoretical predicted measure, which in turn comprises the physiological features that influence the performance, such as gender, height and weight. Accordingly, it has been recently used in the same clinical setting in a cohort of 500 patients, mostly elderly, as the primary outcome to assess physical performance at the end of CR [14]. Moreover, the percent predicted 6MWT value had been previously employed in a research protocol, and it seemed to constitute a better indicator of disease severity in chronic HF patients compared to 6MWT [15]. Further confirmation has been obtained in the context of pulmonary diseases, where respiratory function of patients using non-invasive ventilation for chronic hypercapnic respiratory failure conditions better correlated with percent of predicted 6MWT than actual 6MWT distance [16].

This observational research has been performed in a single CR clinic and is not free from limitations that must be mentioned, with the most important being the single assessment of the study outcome. Due to the contraindications on the administration of 6MWT to patients suffering from recent CV events, both for clinical reasons and presumable reduced reproducibility [12], the test has not been performed at the time of study enrolment. The lack of data on 6MWT at hospital admission precludes to draw its trajectory for each individual in relation to the frailty status. Similarly, CGA was administered only once, upon reaching clinical stability. Moreover, only 30% of women was included in the present research, across all groups. This imbalance in the composition and features of the study population, although in line with the current literature [37], can certainly represent a limitation of the study; nevertheless, the proposed model presents comparable results by analyzing the two genders separately (data not shown).

### Conclusive remarks

FI resulted to be significantly and independently correlated to 6MWT ratio in a population of elderly patients undergoing CR. Frailty is a multifactorial geriatric syndrome whose assessment requires CGA and is essential for prognostic evaluation of older patients, also in CR clinical setting. Further studies are needed to establish the best strategies to assess frailty and physical function in CR setting, in order to incorporate them within the workflows of rehabilitation clinics and target specific geriatric deficits that would interfere with the success of training programs.

## Supplementary Information

Below is the link to the electronic supplementary material.

Supplementary file1 (DOCX 16 KB)

## Data Availability

The datasets generated during and/or analyzed during the current study are available from the corresponding author on reasonable request.

## References

[1] Eurostat (2020). Ageing Europe - Looking at the lives of older people in the EU.

[2] WHO (2017). Integrated care for older people Guidelines on community-level interventions to manage declines in intrinsic capacity. Integrated care for older people: guidelines on community-level interventions to manage declines in intrinsic capacity.

[3] Clegg A, Young J, Iliffe S (2013). Frailty in elderly people. Lancet.

[4] Fried LP, Tangen CM, Walston J (2001). Frailty in older adults: evidence for a phenotype. J Gerontol A Biol Sci Med Sci.

[5] Rockwood K, Song X, MacKnight C (2005). A global clinical measure of fitness and frailty in elderly people. CMAJ.

[6] Kulminski AM, Ukraintseva SV, Kulminskaya IV (2008). Cumulative deficits better characterize susceptibility to death in elderly people than phenotypic frailty: lessons from the cardiovascular health study. J Am Geriatr Soc.

[7] Xue QL, JingTian WJD (2020). Discrepancy in frailty identification: move beyond predictive validity. J Gerontol A Biol Sci Med Sci.

[8] Abbafati C, Abbas KM, Abbasi-Kangevari M (2020). Global burden of 369 diseases and injuries in 204 countries and territories, 1990–2019: a systematic analysis for the Global Burden of Disease Study 2019. Lancet.

[9] Ss V, A A, HJ A,  (2021). Heart disease and stroke statistics-2021 update a report from the american heart association. Circulation.

[10] Giallauria F, Vigorito C, Tramarin R (2010). Cardiac rehabilitation in very old patients: data from the Italian survey on cardiac rehabilitation-2008 (isyde-2008)–official report of the Italian association for cardiovascular prevention, rehabilitation, and epidemiology. J Gerontol A Biol Sci Med Sci.

[11] Searle SD, Mitnitski A, Gahbauer EA (2008). A standard procedure for creating a frailty index. BMC Geriatr.

[12] Crapo RO, Casaburi R, Coates AL (2002). ATS statement: Guidelines for the six-minute walk test. Am J Respir Crit Care Med.

[13] Enrichi PL, Sherrill DL (1998). Reference equations for the six-minute walk in healthy adults. Am J Respir Crit Care Med.

[14] Cañas F, Giraldo GC, Murillo A (2021). Benefits of cardiac rehabilitation on functional status and mood disorders in elderly and very elderly patients: a prospective cohort sTUDY. J Cardiopulm Rehabil Prev.

[15] Balashov K, Feldman DE, Savard S (2008). Percent predicted value for the 6-minute walk test: using norm-referenced equations to characterize severity in persons with CHF. J Card Fail.

[16] Güngör G, Karakurt Z, Adigüzel N (2013). The 6-minute walk test in chronic respiratory failure: does observed or predicted walk distance better reflect patient functional status?. Respir Care.

[17] Buttery AK (2020). Cardiac rehabilitation for frail older people. Adv Exp Med Biol.

[18] Thomas RJ, Balady G, Banka G (2018). 2018 ACC/AHA clinical performance and quality measures for cardiac rehabilitation: a report of the american college of cardiology/american heart association task force on performance measures. J Am Coll Cardiol.

[19] British Heart Foundation (2018) National Audit of Cardiac Rehabilitation (NACR) Report 2018 - BHF. In: Natl. Audit Card. Rehabil. Qual. Outcomes Rep. 2018. https://www.bhf.org.uk/informationsupport/publications/statistics/national-audit-of-cardiac-rehabilitation-quality-and-outcomes-report-2018. Accessed 7 Dec 2021

[20] Gielen S, Simm A (2017). Frailty and cardiac rehabilitation: a long-neglected connection. Eur J Prev Cardiol.

[21] Bencivenga L, Grieco FV, Femminella GD (2017). Management and treatment of cardiovascular diseases in the elderly. Curr Pharmacogenomics Pers Med.

[22] Singh M, Stewart R, White H (2014). Importance of frailty in patients with cardiovascular disease. Eur Heart J.

[23] Vigorito C, Abreu A, Ambrosetti M (2017). Frailty and cardiac rehabilitation: a call to action from the EAPC Cardiac rehabilitation section. Eur J Prev Cardiol.

[24] Vahanian A, Beyersdorf F, Praz F (2021). 2021 ESC/EACTS guidelines for the management of valvular heart disease. Eur Heart J.

[25] Graham MM, Galbraith PD, O’Neill D (2013). Frailty and outcome in elderly patients with acute coronary syndrome. Can J Cardiol.

[26] Ekerstad N, Swahn E, Janzon M (2014). Frailty is independently associated with 1-year mortality for elderly patients with non-ST-segment elevation myocardial infarction. Eur J Prev Cardiol.

[27] White HD, Westerhout CM, Alexander KP (2016). Frailty is associated with worse outcomes in non-st-segment elevation acute coronary syndromes: insights from the targeted platelet inhibition to clarify the optimal strategy to medically manage acute coronary syndromes (trilogy acs) trial. Eur Hear j Acute Cardiovasc care.

[28] McNallan SM, Singh M, Chamberlain AM (2013). Frailty and healthcare utilization among patients with heart failure in the community. JACC Heart Fail.

[29] McNallan SM, Chamberlain AM, Gerber Y (2013). Measuring frailty in heart failure: a community perspective. Am Heart J.

[30] Kitzman DW, Whellan DJ, Duncan P (2021). Physical rehabilitation for older patients hospitalized for heart failure. N Engl J Med.

[31] Fonteles Ritt L, Oliveira ME, F, Santos Pereira Ramos J,  (2021). Impact of a cardiovascular rehabilitation program on frailty indicators in elderly patients with heart disease. Eur J Prev Cardiol.

[32] Kehler DS, Giacomantonio N, Firth W (2020). Association between cardiac rehabilitation and frailty. Can J Cardiol.

[33] Katano S, Hashimoto A, Ohori K (2018). Nutritional status and energy intake as predictors of functional status after cardiac rehabilitation in elderly inpatients with heart failure - a retrospective cohort study. Circ J.

[34] Rao A, Zecchin R, Newton PJ (2020). The prevalence and impact of depression and anxiety in cardiac rehabilitation: a longitudinal cohort study. Eur J Prev Cardiol.

[35] Dracup K (1994). Cardiac rehabilitation the role of social support in recovery and compliance. Soc Support Cardiovasc Dis.

[36] Pogosova N, Saner H, Pedersen SS (2015). Psychosocial aspects in cardiac rehabilitation: from theory to practice. a position paper from the cardiac rehabilitation section of the european association of cardiovascular prevention and rehabilitation of the european society of cardiology. Eur J Prev Cardiol.

[37] Feola M, Garnero S, Daniele B (2015). Gender differences in the efficacy of cardiovascular rehabilitation in patients after cardiac surgery procedures. J Geriatr Cardiol.

