# Supplementary material for: Role of frailty on cardiac rehabilitation in hospitalized older patients
Source: Aging Clin Exp Res. 2022 Sep 5;34(11):2675–82. doi: 10.1007/s40520-022-02220-x (PMC9675689; doi:10.1007/s40520-022-02220-x)
Supplement: Supplementary file 1 — Supplementary file1 (DOCX 16 KB) [file 40520_2022_2220_MOESM1_ESM.docx]

Supplemental Materials

| **List of 40 Variables included in the frailty index** | **Cut Point** |
| --- | --- |
| Help Bathing | Yes = 1, No = 0 |
| Help Dressing | Yes = 1, No = 0 |
| Help getting in/out of Chair | Yes = 1, No = 0 |
| Help Walking around house | Yes = 1, No = 0 |
| Help Eating | Yes = 1, No = 0 |
| Help Grooming | Yes = 1, No = 0 |
| Help Using Toilet | Yes = 1, No = 0 |
| Help up/down Stairs | Yes = 1, No = 0 |
| Help lifting 10 lbs | Yes = 1, No = 0 |
| Help Shopping | Yes = 1, No = 0 |
| Help with Housework | Yes = 1, No = 0 |
| Help with meal Preparations | Yes = 1, No = 0 |
| Help taking Medication | Yes = 1, No = 0 |
| Help with Finances | Yes = 1, No = 0 |
| Lost more than 10 lbs in last year | Yes = 1, No = 0 |
| Self Rating of Health | Poor = 1, Fair = 0.75, Good = 0.5, Very Good = 0.25, Excellent = 0 |
| How Health has changed in last year | Worse = 1, Better/Same = 0 |
| Stayed in Bed at least half the day due to health (in last month) | Yes = 1, No = 0 |
| Cut down on Usual Activity (in last month) | Yes = 1, No = 0 |
| Walk outside | <3 days = 1, ≤ 3 days = 0 |
| Feel Everything is an Effort | Most of time = 1, Sometime = 0.5, Rarely = 0 |
| Feel Depressed | Most of time = 1, Sometime = 0.5, Rarely = 0 |
| Feel Happy | Most of time = 0, Sometime = 0.5, Rarely = 1 |
| Feel Lonely | Most of time = 1, Sometime = 0.5, Rarely = 0 |
| Have Trouble getting going | Most of time = 1, Sometime = 0.5, Rarely = 0 |
| High blood pressure | Yes = 1, Suspect = 0.5, No = 0 |
| Heart attack | Yes = 1, Suspect = 0.5, No = 0 |
| CHF | Yes = 1, Suspect = 0.5, No = 0 |
| Stroke | Yes = 1, Suspect = 0.5, No = 0 |
| Cancer | Yes = 1, Suspect = 0.5, No = 0 |
| Diabetes | Yes = 1, Suspect = 0.5, No = 0 |
| Arthritis | Yes = 1, Suspect = 0.5, No = 0 |
| Chronic Lung Disease | Yes = 1, Suspect = 0.5, No = 0 |
| MMSE | <10 = 1, 11–17 = 0.75, 18–20 = 0.5, 20–24 = 0.25, >24 = 0 |
| Risk for falls | POMA < 19 = 1, 19-24 = 0.5, 25-28 = 0 |
| Social Support Scale | 1-5 Good: 0, 6-13 Fair: 0.5, 14-17 Low: 1 |
| BMI | See Table S2 |
| Grip Strength | See Table S2 |
| Usual Pace | See Table S2 |
| Rapid Pace | See Table S2 |

Table S1. Health Variables and Cut-Points for the frailty index.
The list of health deficit variables included in the FI and how they were coded as deficit (modified from Searle SD, et al. *BMC Geriatr* 2008).

| **Variable** | **Deficit for Men** | **Deficit for Women** |
| --- | --- | --- |
| Body Mass Index (BMI) | <18.5, ≥ 30 as a deficit. | <18.5, ≥ 30 as a deficit. |
|  | 25-<30 as a 'half deficit' | 25-<30 as a 'half deficit' |
| Grip Strength (GS in kg) | <27 | <16 |
| Rapid pace Walk (sec) | >10 | >10 |
| Usual pace Walk (sec) | >16 | >16 |

Table S2. Deficit cut off values for continuous variables by sex and source of cut off (modified from Searle SD, et al. *BMC Geriatr* 2008).
